# Urological surgical robots in China: state of the art and future prospects

**DOI:** 10.1097/JS9.0000000000001377

**Published:** 2024-03-25

**Authors:** Aimin Jiang, Ying Liu, Chen Cai, Peng Luo, Linhui Wang

**Affiliations:** a Department of Urology, Changhai Hospital, Naval Medical University (Second Military Medical University); b Department of Special Clinic, Changhai Hospital, Naval Medical University (Second Military Medical University), Shanghai; c Departmen of Oncology, Zhujiang Hospital, Southern Medical University, Guangzhou, People’s Republic of China

*Dear Editor,*

Robotic-assisted surgery represents a significant evolution in operative methodologies, following the advancements from open to laparoscopic interventions, and is considered the third major innovation in surgical practices. As of the current date, a variety of robotic surgical systems have garnered approval for clinical application, including but not limited to Aesop, Zeus, Da Vinci, and Enhance. Notably, the Da Vinci surgical system, conceived and brought to market by Intuitive Surgical, achieved clearance from the United States Food and Drug Administration (FDA) in the year 2000. This milestone signaled the beginning of the commercial availability of robotic surgical technologies. Through a series of four progressive iterations, the Da Vinci system has ascended to prominence, becoming the most extensively adopted and utilized robotic surgical platform across the globe^1^. The advent of robotic technology in urological surgery has marked a significant milestone in the field of minimally invasive surgery, providing unparalleled precision, dexterity, and visualization while reducing patient recovery times and improving surgical outcomes. A recent publication in the ‘*International Journal of Surgery*’ titled ‘Global trends and prospects in health economics of robotic surgery: a bibliometric analysis’ has garnered our significant interest^2^. This study reports a gradual expansion of robotic surgery systems from urology to orthopedics and thoracic surgery. It also highlights the growing disparity in the adoption of robotic surgery systems between developed and developing countries, suggesting an urgent need to address this gap. As a team dedicated to robotic surgery in urology for many years, I would like to delve into the current state of urological surgical robotics in China and envisage the future prospects of this transformative technology.

In the realm of urology, robotic surgery represents the pinnacle of innovation, combining the benefits of minimally invasive surgery with advanced robotic precision. China, with its rapid technological advancements and commitment to healthcare improvement, has embraced this technology, showcasing significant developments and applications in urological procedures.

The robotic surgical system, epitomized by the da Vinci system, has seen a widespread application in urology, boasting advantages such as enhanced laparoscopic capabilities and minimal invasiveness. In China, the adoption of robotic surgery in urology has been propelled by the development of domestic robotic systems like the KangDuo Surgical Robot-01 system, which has demonstrated feasibility, safety, and efficacy across a range of urological procedures, from partial nephrectomy to radical prostatectomy^3^. Besides, Wang *et al*.^4^ reported that the Shurui system demonstrated safe and effective performance in radical prostatectomy through both transperitoneal and extraperitoneal approaches. Surgical outcomes, tumor control, and urinary continence were favorable. The growth of robotic surgery is also evidenced by the increasing installation of robotic systems across the nation, highlighting the rapid acceptance and integration of this technology in the Chinese healthcare landscape^5^.

Furthermore, the establishment of standardized training programs in robotic surgery aims to elevate the proficiency of surgeons, ensuring the broader application and optimization of robotic surgical techniques^6^. This initiative underscores the importance of a skilled workforce to maximize the potential of robotic surgery in improving patient care.

Looking ahead, the future of robotic urological surgery in China is promising, with ongoing research and development focusing on enhancing the capabilities and accessibility of robotic systems. Innovations in robotic technology, including the development of new robotic platforms tailored for specific urological procedures, are expected to further revolutionize this field. The emphasis on domestic development and innovation is poised to reduce reliance on imported systems, fostering a more sustainable and self-sufficient healthcare technology ecosystem in China.

Moreover, the integration of artificial intelligence and machine learning with robotic surgical systems holds the potential to further enhance surgical precision and outcomes, providing real-time data and predictive analytics to assist surgeons in making informed decisions during procedures^7^.

In general, robot-assisted urological surgery in China stands at a crossroads of opportunity and innovation. With the current advancements and future developments, the country is set to remain at the forefront of adopting and innovating in robotic surgical technologies, ultimately improving the quality of patient care in urology and beyond. The commitment to training, research, and development ensures that the future of robotic surgery in China is not just about maintaining the status quo but about pushing the boundaries of what is possible in surgical care.

## Ethical approval

Not applicable.

## Consent

Not applicable.

## Sources of funding

Not applicable.

## Author contribution

A.J., Y.L., and P.L.: data collection and manuscript preparation; C.C. and L.W.: study concept or design and critical revision and submission.

## Conflicts of interest disclosure

There are no conflicts of interest.

## Research registration unique identifying number (UIN)

Not applicable.

## Guarantor

Linhui Wang.

## Data availability statement

The datasets generated and/or analyzed during the current study are available from the corresponding author on reasonable request.

## Provenance and peer review

Not applicable.
